# Novel word retention in bilingual and monolingual speakers

**DOI:** 10.3389/fpsyg.2014.01024

**Published:** 2014-09-29

**Authors:** Pui Fong Kan, Neeraja Sadagopan

**Affiliations:** Department of Speech, Language, and Hearing Sciences, University of Colorado BoulderBoulder, CO, USA

**Keywords:** bilingualism, bilingual advantage, word learning, word retention, fast mapping

## Abstract

The goal of this research was to examine word retention in bilinguals and monolinguals. Long-term word retention is an essential part of vocabulary learning. Previous studies have documented that bilinguals outperform monolinguals in terms of retrieving newly-exposed words. Yet, little is known about whether or to what extent bilinguals are different from monolinguals in word retention. Participants were 30 English-speaking monolingual adults and 30 bilingual adults who speak Spanish as a home language and learned English as a second language during childhood. In a previous study (Kan et al., 2014), the participants were exposed to the target novel words in English, Spanish, and Cantonese. In this current study, word retention was measured a week after the fast mapping task. No exposures were given during the one-week interval. Results showed that bilinguals and monolinguals retain a similar number of words. However, participants produced more words in English than in either Spanish or Cantonese. Correlation analyses revealed that language knowledge plays a role in the relationships between fast mapping and word retention. Specifically, within- and across-language relationships between bilinguals' fast mapping and word retention were found in Spanish and English, by contrast, within-language relationships between monolinguals' fast mapping and word retention were found in English and across-language relationships between their fast mapping and word retention performance in English and Cantonese. Similarly, bilinguals differed from monolinguals in the relationships among the word retention scores in three languages. Significant correlations were found among bilinguals' retention scores. However, no such correlations were found among monolinguals' retention scores. The overall findings suggest that bilinguals' language experience and language knowledge most likely contribute to how they learn and retain new words.

## Introduction

The purpose of this study was to examine word retention in bilinguals and monolinguals. Learning a new spoken word involves not only the acquisition of linguistic information about a word during the initial encounters but also the retention of the word in the long-term (e.g., Markson and Bloom, 1997; Wilkinson and Mazzitelli, 2003; Capone and McGregor, 2005; Horst and Samuelson, 2008; Kan and Kohnert, 2012; Kucker and Samuelson, 2012; Kan, 2014). Previous studies have documented that bilinguals outperform monolinguals in the immediate retrieval of newly-exposed novel words (e.g., Kaushanskaya and Marian, 2009; Kaushanskaya and Yoo, 2011; Kaushanskaya and Rechtzigel, 2012; Kaushanskaya, 2012). Unlike cognitive skills (e.g., executive functions, attention, and working memory) that are associated with immediate word learning and retrieval in bilinguals (e.g., Bialystok, 1999; Bialystok and Martin, 2004; Bialystok et al., 2006; Kaushanskaya and Marian, 2009; Kaushanskaya, 2012), the cognitive skills that are related to long-term word retention are less understood. According to the revised hierarchical model (Kroll and Stewart, 1994; Kroll et al., 2010), lexical connections between the two languages in bilinguals are linked to common conceptual memory representations; and the connections to conceptual memory depend on bilinguals' L1 and L2 experiences and knowledge. In the same vein, evidence regarding bilinguals' language knowledge in two languages, bilinguals' episodic memory, and their semantic memory (e.g., Francis, 1999; Kormi-Nouri et al., 2003, 2008, 2012; Sheng et al., 2006, 2013; Bialystok and Feng, 2009) suggests that bilinguals differ from monolinguals in how they encode, retain, and organize words.

In experimental settings, the measure of spoken word retention involves an initial stage of novel word exposure and a long exposure-retrieval interval ranging from 24 h to several months, different from the immediate retrieval in fast mapping tasks (e.g., Rice et al., 1994; Markson and Bloom, 1997; Wilkinson and Mazzitelli, 2003; Horst and Samuelson, 2008; Kan, 2014). Many word learning studies use novel words—i.e., non-words that follow the phonological rules of a particular language but do not carry any meanings—as stimuli in order to control for previous and ongoing experiences with the target words. The present study examined bilinguals' novel word retention with a one-week exposure-retrieval interval. This current study was a follow-up study of Kan et al. (2014) in which Spanish-English bilinguals outperformed English-speaking monolinguals in identifying newly-exposed novel words not only in English but also in a language foreign to both groups (i.e., Cantonese). Importantly, Kan et al. (2014) found there to be speech practice effects on fast mapping production for both bilinguals and monolinguals. Specifically, speech practice involved the participants' listening and repeating of target word-forms prior to their undertaking the fast mapping task. The present follow-up study focuses on factors that are associated with word retention in bilinguals and monolinguals. It aims at understanding the relationships between bilinguals' language learning experience, their speech practice, and their vocabulary learning.

Successfully retaining new words requires a complex learning mechanism that interacts with the encountering of new words. Of interest in this current study was the impact of bilinguals' dual language experience on word retention, given the bilingual advantages for immediately retrieving newly-exposed novel words, as pointed out in a previous study (see Kan et al., 2014). According to Baddeley's working memory model (Baddeley et al., 1998), the initial acquisition of newly-encountered words relies on a cognitive system called the phonological loop, which is responsible for temporarily storing new phonological word-forms and for transferring short-term memory traces into long-term memory. This model suggests that the temporarily stored word knowledge (e.g., in a fast mapping task) is associated with how words are stored for long term. Given bilinguals' unique cognitive functions for forming initial word representations as a result of their bilingual experience (e.g., Kaushanskaya, 2012), one might hypothesize that bilinguals outperform monolinguals in retaining newly-exposed words. Consistent with this hypothesis, Kan (2014) found that Hmong-English bilingual preschool children's fast mapping performance in L1 and in L2 was positively linked to the number of words retained in English (L2). Indeed, some studies have shown that bilingual experience tends to lead to stronger episodic and semantic memory as measured by a variety of tasks such as fluency tasks and free recall tasks (e.g., Kormi-Nouri et al., 2003, 2008).

However, previous studies that examined bilinguals' vocabulary acquisition and their word retrieval skills suggest that bilinguals tend not to outperform monolinguals in retrieving learned words (e.g., Bialystok, 2009; Bialystok et al., 2010; Festman, 2012). For example, bilingual children have smaller receptive and expressive vocabularies and slower vocabulary growth rate in each language than do their monolingual peers (e.g., Windsor and Kohnert, 2004; Oller et al., 2007; Vagh et al., 2009; Bialystok et al., 2010; Thordardottir, 2011; Bialystok and Luk, 2012; Poulin-Dubois et al., 2013). Yet, many studies point out that bilingual children have conceptual vocabulary similar to that of their monolingual peers (e.g., Pearson et al., 1993; Bedore et al., 2005). Consistently, previous studies found that bilingual children, as well as adults, have slower reaction times than do monolinguals on picture naming tasks (e.g., Windsor and Kohnert, 2004; Rodriguez-Fornells et al., 2005; Gollan et al., 2005) and demonstrate poorer performance on verbal fluency tasks (e.g., Sandoval et al., 2010; Kormi-Nouri et al., 2012; Festman, 2012). Taken together, these considerations indicate that the advantage that is observed in fast mapping might not persist at the level of long-term word retrieval.

Several factors have been proposed to explain bilinguals' poorer performance in retrieving retained words. One factor is bilinguals' infrequent use and activation of each language as compared to monolinguals' use (e.g., Gollan et al., 2008). Another factor is the competition that exists between the two languages during retrieval (e.g., Hernandez et al., 2005; Sandoval et al., 2010; Bialystok and Craik, 2010; Jones et al., 2012; Kroll et al., 2012). Indeed, several internal and external factors (e.g., language proficiency, input, opportunities to use the words, semantic organization) could contribute to the retention of word knowledge (e.g., Rice et al., 1994; Thorn et al., 2002; Kormi-Nouri et al., 2003; Thorn and Frankish, 2005; Van Geert, 2008; Francis and Gutierrez, 2012; Francis et al., 2014; Kan, 2014). For example, bilingual children appear to learn and retain more new words in their stronger language than in their weaker language (e.g., Kan and Kohnert, 2012; Kan et al., 2014; Kan, 2014). In this current study, we explored whether strengthened fast mapping performance through speech practice would lead to better word retention. Of interest was whether bilinguals retained more words with speech practice, given their bilingual experience and cognitive functions (e.g., executive functions, working memory) that are described earlier. In addition, we examined whether bilinguals' existing language knowledge in two languages contributes to word retention, given the role of language knowledge in word encoding, storing, and retrieval (e.g., McGregor and Waxman, 1998; Thorn et al., 2002; Thorn and Frankish, 2005; Luo et al., 2010; Gray and Brinkley, 2011).

Models of language acquisition suggest a dynamic relationship between input, lexical organization, and word learning (e.g., van Geert, 1998; Borovsky and Elman, 2006; Li et al., 2007; Li, 2009). Consistently, the results from empirical studies also demonstrate that bilinguals' interactive L1–L2 language system (e.g., Francis, 1999; Su, 2001; Gildersleeve-Neumann et al., 2009; Kan and Kohnert, 2012; Kan, 2014) is associated with their word learning performance (e.g., Bialystok et al., 2005b; Sheng et al., 2006; Marchman et al., 2010; Kan and Kohnert, 2012; Kan, 2014). A relevant consideration for this present study was the effect of bilinguals' existing language knowledge in two languages on their ability to process incoming input and to retain new word knowledge. According to the revised hierarchical model (Kroll and Stewart, 1994), bilinguals' experience and knowledge in L1 and L2 play an important role in the connections between the word-form lexicons for L1 and L2 and the shared conceptual store. Consistent with this framework, two aspects of bilingual language processing might be related to the complex relationships between language knowledge and word retention. First, there is evidence indicating that processing information in a weaker language tends to place greater demands on cognitive resources (e.g., Francis and Gutierrez, 2012; Kan et al., 2014). Accordingly, greater demands for processing new words in an unfamiliar language might lead to relatively weaker representations of the target words during the initial fast mapping tasks. Second, one important aspect of fast mapping and word learning experiments is to reveal the learners' knowledge about the link between a word form and its referent. Learning a new concept in two languages requires learning the links to two word forms in two languages. Previous findings suggest that words in an unfamiliar language tend to be weakly linked to the referent, as compared to words in a strong language (e.g., Dufour and Kroll, 1995; Dong et al., 2005; Misra et al., 2012). That is, the newly-learned word-referent associations in bilinguals and monolinguals are likely to be weaker for words in a foreign language (i.e., in this case, Cantonese; e.g., van Hell and Candia Mahn, 1997); and bilinguals' language knowledge in each language might contribute to the strength of the word-referent links for each language. However, such cognitive demands and weak links could be reduced by elaborative processing during the presentation phase and by scaffolding strategies during retrieval (e.g., Kiernan and Gray, 1998; Capone and McGregor, 2005; Francis and Gutierrez, 2012).

The goal of this present research was to examine whether there are differences between bilinguals and monolinguals in novel word retention. The current study was a follow-up study that examined the retention of words that were previously learned in a fast mapping task (see the results in Kan et al., 2014). In particular, Kan et al. (2014) examined the effects of speech practice on fast mapping performance. The participants in the two speech practice groups were required to repeat all target novel words that were randomly presented (15 times for the intensive practice group and 5 times for the less intensive practice group) before the fast mapping task. Word retention was measured a week after the fast mapping task. There were four specific research questions posed in this current study:

1. Does initial speech practice have a positive effect on word retention? Do bilinguals retain more words with speech practice than do monolinguals, given bilinguals' unique experience and cognitive functions?
2. Are there any differences between monolinguals and bilinguals in retaining words in their familiar language(s) (English; English and Spanish) and in a language foreign to both groups (i.e., Cantonese)?
3. Are there any correlations between participants' word retention and their initial fast mapping performance (data from Kan et al., 2014)? If so, are there any differences between bilinguals and monolinguals?
4. Do participants who retain more words in one language also retain more words in another language? If so, are there any significant differences in this regard between bilinguals and their monolingual peers?

We expected that participants in both monolingual and bilingual groups would demonstrate a speech practice advantage. That is, participants who experienced intensive speech practice would produce a greater number of words than the subset of participants who underwent less intensive speech practice or than those in the control group. However, as described earlier, many internal and external factors could affect the patterns of long-term word retention of bilinguals and monolinguals. If rehearsal prevents the decay of the initial representations (e.g., Baddeley et al., 1998; Kaushanskaya and Yoo, 2011), it is possible that speech practice could contribute to forming the initial word representations, leading to stronger word retention. However, it is also likely that speech practice does not have an effect on word retention when no additional input is available during the exposure-retrieval interval. In addition, we also anticipated that language knowledge would play an important role in word retention. Of interest in this study were the patterns of word retention in bilinguals and monolinguals. Previous studies have demonstrated that bilinguals differ from monolinguals in cognitive functions such as executive functions or such as phonological memory (e.g., Kaushanskaya and Marian, 2009; Kaushanskaya, 2012). In Kan et al. (2014), a bilingual advantage in fast mapping performance was found for comprehension scores across the three speech conditions. If language knowledge and strong fast mapping performance are the foundation for word retention (cf. Baddeley et al., 1998; Thorn et al., 2002), it is possible that the bilingual advantage persists at the level of long-term word retention. However, the bilingual advantage might not persist after 1 week, given the complex relationships between word retention and other factors such as additional input and language proficiency (e.g., Thorn et al., 2002; Luo et al., 2010). In addition, given the evidence showing the role of language knowledge and experience in word retention (e.g., van Hell and Candia Mahn, 1997; Kan, 2014), we expected that both monolingual and bilingual groups would retain fewer words in Cantonese (the control language).

## Methods

Participants were 60 young adults (*M* = 23.03 years; *SD* = 2.99). All of them had participated in the experiment reported in Kan et al. (2014). Half of them were English-speaking monolinguals and half were Spanish-English bilinguals who learned Spanish as a home language and English as a second language during childhood. On average, these bilingual participants had 15.83 years of education during which they had 14.68 years of education in Spanish or in both Spanish and English. All bilingual participants reported that they used both Spanish and English functionally. In particular, they reported that they used Spanish while speaking with family and friends in home settings, whereas they used English more often in academic and work settings. In Kan et al. (2014), the monolingual and bilingual participants were randomly assigned to three groups (*n* = 10 per group): intensive speech practice (15 repetitions), moderate speech-training (5 repetitions), and control groups (no repetition). Table 1 summarizes the characteristics of the participants across the 6 groups. Participants' vocabulary knowledge in English and in Spanish (for bilinguals) was measured using two Woodcock-Muñoz (WM) subtests: picture vocabulary and verbal analogy. Their nonverbal intelligence was measured using the Test of Nonverbal Intelligence (TONI). A 2 × 3 Analysis of Variance Tests (ANOVAs) with language groups (Monolingual vs. Bilingual) and experimental-control group (the 2 experimental groups and the control group) as between-subject independent variables showed that the six groups exhibited no significant age-related differences [Language group: *F*_(1, 54)_ = 0.64, *p* > 0.05; Speech training group: *F*_(1, 54)_ = 2.04, *p* > 0.05]. Nor were they significantly different in terms of their TONI scores [Language group: *F*_(1, 54)_ = 2.8, *p* > 0.05; Speech training group: *F*_(1, 54)_ = 2.55, *p* > 0.05], their WM Verbal Analogies scores [Language group: *F*_(1, 54)_ = 0.73, *p* > 0.05; Speech training group: *F*_(1, 54)_ = 0.3, *p* > 0.05], or their years of education [Language group: *F*_(1, 54)_ = 0.27, *p* > 0.05; Speech training group: *F*_(1, 54)_ = 1.6, *p* > 0.05]. However, the 30 monolingual participants, as a group, had significantly higher WM Picture Vocabulary scores in English than did the 30 bilingual participants [Language group: *F*_(1, 54)_ = 10.72, *p* < 0.01]. In addition, there were no significant differences among the three groups of bilingual participants in the WM picture vocabulary scores in Spanish [*F*_(1, 27)_ = 2.76, *p* > 0.05] or in the WM verbal analogy scores in Spanish [*F*_(1, 27)_ = 2.01, *p* > 0.05].

**Table 1:** **Participant description information**.

**Groups**	**Monolingual**			**Bilingual**		
	**Experimental Group 1 15 repetitions**	**Experimental Group 2 5 repetitions**	**Control Group 0 repetitions**	**Experimental Group 1 15 repetitions**	**Experimental Group 2 5 repetitions**	**Control Group 0 repetitions**
	**Mean (SD**)	**Mean (SD**)	**Mean (SD**)	**Mean (SD**)	**Mean (SD**)	**Mean (SD**)
Age	21.1 (1.8)	21.4 (1.08)	22 (1.7)	22.4 (3)	22.4 (2.51)	22.4 (3.33)
Years of education	15.5 (2.5)	16.1 (2.24)	16.33 (2.31)	15.9 (2.68)	13.33 (2.68)	17.91 (2.68)
TONI	96.3 (7)	100.3 (5.1)	105.9 (9.76)	102.3 (13.71)	95.6 (8.4)	100.5 (11.08)
WMEV	45 (2.87)^*^	46.1 (4)^*^	46.44 (1.94)^*^	44.8 (5.03)	42.5 (3.95)	42.8 (4.8)
WMEA	28.2 (3.55)	29.2 (2.2)	28.75 (3.06)	29.1 (3.51)	29.1 (3.78)	26.4 (5.8)
WMSV	−	−	−	45.6 (4.77)	42.1 (7.25)	44 (2.98)
WMSA	−	−	−	33.56 (2.83)	29.4 (4.74)	29.2 (8.01)

*TONI, TONI standard score; WMEV, Woodcock-Muñoz English picture vocabulary score; WMEA, Woodcock- Muñoz Spanish analogy score; WMSV, Woodcock-Muñoz Spanish picture vocabulary score; WMSA, Woodcock- Muñoz Spanish analogy score*.

* *p <0.05*.

Stimuli that were used in Kan et al. (2014) and in current study were 16 novel objects each of which was paired with one novel word in English, one in Spanish, and one in Cantonese (i.e., a single novel object was paired with three phonologically distinct word forms). The novel words were nonwords that were based on phonological rules of that respective language and that did not carry any meanings. Each novel word in Cantonese carried a lexical tone. There were a total of 48 novel word forms across the three languages.

There were two phases in Kan et al. (2014): a speech practice phase and a fast mapping phase. During the speech practice phase, the participants of the two experimental groups were asked to listen and to repeat the 16 target novel words in each language (15 repetitions or 5 repetitions). No novel objects were presented during this phase. Immediately after speech practice, participants from the two experimental groups participated in the fast mapping task. Participants from the control group, who did not receive speech practice, participated in the fast mapping task at the beginning of the session. During the fast mapping task, auditory models of the novel words, along with the novel objects, were presented on a computer. The 16 novel words were presented in 4 blocks (i.e., four novel words per block). The order of the 3 languages and the order of the 4 blocks were counterbalanced. The fast mapping task for each block for each language involved two phases: a presentation phase and a probing phase. During the presentation phase, each participant was presented with each novel image two times along with the auditory word-form label. At the end of each block, each participant's novel word knowledge was measured using a novel word production task, which was followed by a novel word comprehension task. In the production probe, participants were asked to name each of the target objects. No prompts or feedback was given. In the comprehension probe, participants were asked to identify the target object from an array of novel objects. As exhibited in Table 2, the results showed a significant speech practice effect on the fast mapping production scores but not on the fast mapping comprehension scores. Bilinguals outperformed monolinguals on the fast mapping comprehension task but not on the fast mapping production task.

**Table 2:** **Monolingual and bilingual participants' word retention performance across experimental and control groups**.

**Groups**	**Monolingual (***n*** = **30**)**			**Bilingual (***n*** = **30**)**		
	**Experimental Group 1 15 repetitions**	**Experimental Group 2 5 repetitions**	**Control Group 0 repetitions**	**Experimental Group 1 15 repetitions**	**Experimental Group 2 5 repetitions**	**Control Group 0 repetitions**
	**Mean (SD)**	**Mean (SD)**	**Mean (SD)**	**Mean (SD)**	**Mean (SD)**	**Mean (SD)**
English production	1.4 (2.01)	1 (1.41)	0.7 (0.68)	1.5 (1.96)	1.2 (1.14)	0.6 (.967)
English comprehension	7.4 (3.34)	6.9 (1.66)	7.2 (3.77)	7.8 (3.49)	8 (2.92)	5.7 (2.26)
Spanish production	0.5 (0.97)	0.2 (0.42)	0.3 (0.48)	1.1 (1.66)	0.7 (0.68)	0.3 (0.48)
Spanish comprehension	5.9 (2.69)	5.7 (1.89)	5.4 (2.27)	7.8 (3.43)	6.89 (1.62)	6.1 (2.6)
Cantonese production	0.1 (0.32)	0.4 (0.52)	0.4 (0.97)	0.5 (0.97)	0.4 (0.52)	0 (0)
Cantonese comprehension	6.3 (2.06)	7 (1.56)	5.5 (1.9)	5.3 (2.11)	7.67 (4)	5.8 (1.87)

*The maximum score for each task in each language was 16*.

### Word retention task

In this current study the number of retained novel words in English, Spanish, and Cantonese was measured a week after the fast mapping task, using the same novel word production and comprehension probes (Kan et al., 2014). Each participant was not exposed to the novel words or the novel images during the one-week interval. At the beginning of the word retention task, each participant was asked to name the 16 novel objects in each target language. Then he/she was asked to identify the target object from an array of 4 novel objects. The order of language was counterbalanced. No prompts or feedback were given during either task.

The production tasks were scored by trained research assistants, who are native speakers of Spanish and/or English, as well as by the first author, who is a native speaker of Cantonese. For the production task under the English and the Spanish conditions, the respective participant received one point when he/she verbally produced the non-word exactly as it was presented during the presentation phase of the fast mapping task in Kan et al. (2014). For the control language condition (i.e., Cantonese), participants were required to pronounce all *phonemes* correctly. Incorrect production of *tones* was not counted as incorrect, in order to keep scoring criteria and procedures comparable across the three language conditions. For the comprehension probes, each participant received one point when he/she correctly identified the target object. If the participants did not respond at all, or did not respond during the allotted time (i.e., 8000 ms), the answer was marked as incorrect. Ten percent of the production data and the comprehension data were re-examined by another examiner. The inter-rater reliability was 0.9 for the production probes and 0.98 for the comprehension probes.

## Results

Table 2 summarizes the word retention results for the bilingual and monolingual groups. On average, the production retention scores across all groups were very low for all language conditions (mean scores: range from 0 to 1.5 out of 16). The comprehension retention scores were above chance for all language conditions [English: *t*_(59)_ = 17.89, *p* < 0.001; Spanish: *t*_(59)_ = 18.36, *p* < 0.001; Cantonese: *t*_(59)_ = 19.2, *p* < 0.001], indicating that the participants as a group retained some knowledge of the novel words after one week.

### Speech practice, bilingual experience, and word retention

A repeated measures MANOVA was used to examine the word retention scores—with speech practice (i.e., as regards 2 experimental groups and a control group) and bilingual experience (i.e., monolingual vs. bilingual) serving as between-subject independent variables, and with language (i.e., English, Spanish, and Cantonese) serving as a within-subject independent variable. The results are summarized in Table 3. There were no significant effects of speech practice or bilingual language experience on the word retention comprehension scores or production scores, and there were no interactions between monolingual-bilingual group and speech practice. These findings suggest that bilinguals and monolinguals retain a similar number of words across the speech practice conditions. However, there was a within-subject language effect on the comprehension scores; but there were no significant interactions between language and bilingual experience or between language and speech practice conditions. Multiple comparisons also showed that participants correctly identified more words in English than words in the other two languages. These findings suggest that, after a one-week interval, monolinguals and bilinguals retain more novel words in English than in Spanish or in Cantonese.

**Table 3:** **Effects of speech practice, bilingual language experience, and language on word retention**.

	***df***	***F***	***p***
**COMPREHENSION**			
Monolingual-bilingual group	1	0.72	0.40
Speech practice	2	1.71	0.19
Language	2	3.10	0.049^*^
Group × speech practice	2	0.45	0.64
Language × monolingual-bilingual group	2	1.58	0.21
Language × speech practice	4	1.22	0.31
Language × monolingual-bilingual group × speech practice	4	0.97	0.43
**PRODUCTION**			
Monolingual-bilingual group	1	0.58	0.45
Speech practice	2	1.97	0.15
Language	2	11.23	0^***^
Group × speech practice	2	0.70	0.50
Language × monolingual-bilingual group	2	0.83	0.44
Language × speech practice	4	0.80	0.53
Language × monolingual-bilingual group × speech practice	4	0.20	0.94

* p < 0.05;

*** *p < 0.001*.

Given the speech practice effects were found on the fast mapping production scores in Kan et al. (2014), we explored whether there were speech practice effects on word retention production scores (see Table 3). Results showed that there were no significant effects of speech practice [*F*_(2, 54)_ =.59, *p* > 0.05] or bilingual language experience [*F*_(1, 54)_ = 0.66, *p* > 0.05] on the word retention production scores, and there were no interactions between monolingual-bilingual group and speech practice [*F*_(2, 54)_ = 0.53, *p* > 0.05].

### Fast mapping performance and word retention in bilinguals and monolinguals

One central question in this study was to examine whether fast mapping performance served as a foundation for word retention as discussed earlier. In the context of this follow-up study, we focused on whether word retention was associated with strengthened fast mapping performance through speech practice as shown in Kan et al. (2014). Presumably, a wide range of fast mapping scores across the three groups (*n* = 30) allowed us to see this relationship more clearly. Accordingly, correlation analyses were done to examine participant's fast mapping performance (production and comprehension scores) and word retention scores (comprehension scores). Table 4 summarizes the results for the bilinguals and monolinguals.

**Table 4:** **Correlations between fast mapping and word retention in English, Spanish, and Cantonese**.

**Fast mapping (Dataset from Kan et al., 2014)**							
		**Production English**	**Production Spanish**	**Production Cantonese**	**Comprehension English**	**Comprehension Spanish**	**Comprehension Cantonese**
**BILINGUAL PARTICIPANTS**							
Word retention	Comprehension English	0.37^*^	0.35	0.30	0.14	0.13	0.19
	Comprehension Spanish	0.28	0.20	0.16	−0.15	−0.19	−0.15
	Comprehension Cantonese	−0.03	0.04	0.06	0.17	0.21	0.17
**MONOLINGUAL PARTICIPANTS**							
Word retention	Comprehension English	0.26	−0.02	0.22	0.25	0.43^*^	0.28
	Comprehension Spanish	0.08	0.36	0.28	0.04	0.22	−0.14
	Comprehension Cantonese	0.08	−0.25	0.187	0.47^**^	0.21	0.02

*The fast mapping scores are from Kan et al. (2014)*.

* p < 0.05;

** *p < 0.01*.

Correlation analyses showed positive within-language correlations between bilinguals' word retention scores and their fast mapping scores in English. No such within-language correlations were found in Spanish or in Cantonese. These findings suggest that word retention is associated with fast mapping performance in one of the languages that bilinguals are familiar with. For monolinguals, no significant within-language correlations were found between fast mapping and word retention production scores in all three languages. However, there were cross-language relationships between fast mapping Spanish scores and word retention English scores and between fast mapping English scores and word retention Cantonese scores.

### Word retention in english, spanish, and cantonese

Given the floor effects for the production scores, we present only the correlations among the word retention comprehension scores in the three languages. As shown in Table 5, significant correlations were found among bilinguals' retention scores within- and across-languages. For monolinguals, no correlations were found among the retention scores. The findings suggest that bilinguals who retain more words in English also retain more words in Spanish and in Cantonese. Further analyses showed that there were no translation-equivalent links among the retained items across languages in the bilingual group.

**Table 5:** **Bilinguals and monolinguals: The word retention relationships in three languages**.

	**Spanish-English bilinguals**			**Monolinguals**		
	**English Comprehension**	**Spanish Comprehension**	**Cantonese Comprehension**	**English Comprehension**	**Spanish Comprehension**	**Cantonese Comprehension**
English Comprehension	–	0.42*	0.56**	–	0.20	0.22
Spanish Comprehension	0.42*	–	0.10	0.20	–	0.03
Cantonese Comprehension	0.56**	0.10	–	0.22	0.03	–

^*^p < 0.05; ^**^p < 0.01.

## Discussion

This study investigated word retention in bilingual and monolingual young adults. The bilingual participants learned Spanish as a home language from birth and started to learn English as a second language. At the time of testing, the bilingual participants, as a group, reported that they used both Spanish and English functionally. In a previous study (Kan et al., 2014), the same participants were exposed to 16 objects, each of which was paired with one novel word in English, one in Spanish, and one in Cantonese. This current study examined the retention of these target words a week after the exposures. Overall, the results showed that both monolingual and bilingual participants retained similar numbers of target words as measured by a novel word comprehension task. However, bilinguals did not retain more words in English, Spanish, or Cantonese than did monolinguals across all speech practice conditions. This finding is not consistent with the findings regarding bilingual advantage in semantic memory (e.g., Kormi-Nouri et al., 2003).

If bilinguals have an advantage for initial word learning and immediate retrieval (e.g., Kaushanskaya and Marian, 2009; Kan et al., 2014), why did the bilinguals not retain more words than did the monolinguals? One possible explanation of the absence of bilingual effects on word retention is that in addition to fast mapping ability, many factors such as language knowledge, input, and task demands could affect the retention of word knowledge. Indeed, these factors might contribute to the great variability in the number of words retained across groups (see Table 3). Furthermore, according to the Baddeley's working memory model, rehearsal is a key component for long-term retention. In our study, participants were exposed to the novel words during the speech practice and during the fast mapping task a week before the retention task. However, without additional input after the fast mapping, the initial representations might decay because of various factors at the individual levels. Future investigations that examine the effects of input on word retention are needed to verify this explanation. Another explanation is related both to the competition between two languages during retrieval and to the task demands (e.g., Hernandez et al., 2005; Sandoval et al., 2010; Bialystok and Craik, 2010; Jones et al., 2012; Kroll et al., 2012). It is possible that bilinguals outperform monolinguals in retaining the novel words. Nonetheless, the lack-of-bilingual-experience effect is due to the competition between two languages during retrieval. This view is consistent with the complex patterns regarding bilinguals' performance across a variety of tasks. For example, although bilingual children have conceptual vocabulary similar to that of their monolingual peers (e.g., Pearson et al., 1993; Bedore et al., 2005), they tend to be slower on picture naming tasks (e.g., Windsor and Kohnert, 2004) and demonstrate poorer performance on verbal fluency tasks (e.g., Sandoval et al., 2010; Kormi-Nouri et al., 2012).

In addition to word retention comparisons between bilinguals and monolinguals, there were three interesting findings. First, both monolingual and bilingual participants identified more words in English than words in Spanish or words in Cantonese. This finding suggests that language knowledge plays a role in word retention. For monolinguals, poor word retention in Spanish and Cantonese conditions suggests that language experience plays an important role in word retention. One explanation is that processing information in an unfamiliar language requires greater demands on cognitive resources (e.g., Francis and Gutierrez, 2012). Even though monolinguals might be able to form initial word-referent associations, the representations are likely to be weaker for words in a foreign language (in the present case, in Cantonese); and bilinguals' language knowledge in each language might contribute to the strength of the word-referent links for each language. However, this explanation cannot explain bilingual participants' poor word retention in the Spanish conditions. In our sample, the Spanish-English bilingual participants reported that they used Spanish and English functionally and had comparable skills in Spanish and in English as measured by two subtests of Woodcock- Muñoz (WM): picture vocabulary and verbal analogy. It is important to keep in mind that our bilingual participants might use English more often than Spanish across academic and work settings, despite their comparable skills in Spanish and English. It might be that stronger word retention in English suggests shifts in language dominance in favor of English (cf., Kroll et al., 2010).

Second, the initial stage of word learning appears to be associated with the retention of the word knowledge, as suggested by Kan (2014). However, the patterns of such relationships for bilinguals differ from those for monolinguals, suggesting that bilinguals and monolinguals might learn new words differently. Specifically, within-language correlations were found between bilinguals' fast mapping scores and retention scores in English only. However, there were no correlations between bilinguals' fast mapping and word retention in Spanish or in Cantonese. In contrast, we found across-language relationships between monolinguals' English scores and Cantonese and between Spanish and English. The results in the bilingual participants suggest language dominance shifts toward L2 as discussed earlier (cf., Kroll et al., 2010). The results suggest that bilinguals' existing language knowledge in English (e.g., phonological knowledge in English) plays an important role in fast mapping and in word retention. The findings are consistent with the studies that examined the association between young bilingual children's existing vocabulary knowledge (e.g., phonological knowledge) and their skills for learning new words (e.g., Luo et al., 2010; Marchman et al., 2010; Kan and Kohnert, 2012; Kan, 2014). One explanation for this relationship is that existing language knowledge facilitates the encoding of incoming information for temporary storage of initial representations during fast mapping and for later retrieval of the words during the word retention task (Marian and Fausey, 2006; Luo et al., 2010; cf., Francis and Gutierrez, 2012).

In contrast, monolinguals who had better fast mapping performance in English retained more words in Cantonese; and monolinguals who had better fast mapping performance in Spanish retained more English novel words. That is, monolinguals' fast mapping ability in the one language is related to the retaining new words in another language. Monolinguals who learn novel words in Spanish conditions and in Cantonese conditions are similar to early stage L2 learners. According to the revised hierarchical model, during the early stage of L2 learning, there are no direct links between conceptual representations and the L2 words; and learners learn new words in L2 by way of L1. In the learning context of this current study, monolinguals' underlying ability establishing word representations and form-meaning links in English might indicate their skills learning new words in L2. In contrast, our bilingual participants, who have higher proficiency in the L2, they directly establish links between concepts and L2 lexicon without the need to mediate through L1. In order to verify this explanation, more investigations are needed about the word retention and retrieval patterns in bilinguals who have high levels of L1–L2 knowledge.

The third important finding has to do with the patterns of the correlations among the word retention scores across languages in bilinguals and in monolinguals. In particular, bilinguals' retention of novel words in English is correlated with that in Spanish and in Cantonese. Further analyses indicate that there were no translation-equivalent links among the retained items across languages in the bilingual group. That is, the retained lexical forms in the three languages are not linked at the referent level. The correlations simply represent the number of words retained for the three language conditions. By contrast, no such relationships are found in monolinguals' word retention performance. The findings of bilinguals' word retention among the three languages are consistent with previous findings of positive cross-language relationships existing between the two languages in bilingual children (e.g., Ordonez et al., 2002; Bialystok et al., 2005a; Proctor et al., 2006; San Francisco et al., 2006; Branum-Martin et al., 2009). One explanation for the positive cross-language relationships in bilinguals is that bilinguals' two languages are stored and operated in a common system (e.g., Francis, 1999; Dong et al., 2005; Van Geert, 2008) and that their metalinguistic skills are transferred across languages (e.g., Cummins, 1979). Consistent with these views, our data suggest that bilinguals' language experience and language knowledge contribute to a common language learning system that affects word retention in their two languages as well as in a language foreign to them. Accordingly, bilinguals, who have language knowledge in two languages, might be different from monolinguals in terms of how words are retained rather than simply in terms of the number of words retained.

In conclusion, the present study has documented the retention of word knowledge in bilinguals and monolinguals. The results show that bilinguals, who have been found to have an advantage for immediate novel word retrieval, do not retain more words than do monolinguals across all speech practice conditions. However, bilinguals' language knowledge appears to contribute to how words are encoded and retained—a unique characteristic that makes bilinguals differ from monolinguals. These findings provide some information about the relationships between the non-linguistic cognition and language function in bilinguals but yield more questions regarding the mechanisms that underlie bilinguals' learning system. Future studies are needed to pinpoint the interactions between language knowledge and cognitive factors that contribute to how words are encoded, stored, and retrieved in bilinguals.

## Author contributions

Pui Fong Kan was involved in the design of the study and the development of the materials. She was also responsible for collecting data, for analyzing and interpreting the language data, and for preparing of the manuscript. Neeraja Sadagopan was involved in the design of the experiment, particularly focusing on the speech practice effects on fast mapping. She was also involved in interpretation of the speech-related effects and contributed to manuscript preparation.

### Conflict of interest statement

The authors declare that the research was conducted in the absence of any commercial or financial relationships that could be construed as a potential conflict of interest.
